# Modeling Learner Heterogeneity: A Mixture Learning Model With Responses and Response Times

**DOI:** 10.3389/fpsyg.2018.02339

**Published:** 2018-12-05

**Authors:** Susu Zhang, Shiyu Wang

**Affiliations:** ^1^Department of Psychology, University of Illinois at Urbana-Champaign, Urbana, IL, United States; ^2^Quantitative Methodology Program, Department of Educational Psychology, University of Georgia, Athens, GA, United States

**Keywords:** response times, learning behaviors, diagnostic classification model, hidden markov model, mixture model

## Abstract

The increased popularity of computer-based testing has enabled researchers to collect various types of process data, including test takers' reaction time to assessment items, also known as response times. In recent studies, the relationship between speed and accuracy in a learning setting was explored to understand students' fluency changes over time in applying the mastered skills in addition to skill mastery. This can be achieved by modeling the changes in response accuracy and response times throughout the learning process. We propose a mixture learning model that utilizes the response times and response accuracy. Such a model accounts for the heterogeneities in learning styles among learners and may provide instructors with valuable information, which can be used to design individualized instructions. A Bayesian modeling framework is developed for parameter estimation and the proposed model is evaluated through a simulation study and is fitted to a real data set collected from a computer-based learning system for spatial rotation skills.

## 1. Introduction

Educational researchers have shown long term interests in understanding the heterogeneity among online learners. Learners can differ not only in their initial background and general learning ability, but also in terms of how they learn. For example, learners' affects, that is the attitudes, interests, and values that learners exhibit, can influence their behaviors in the learning process and hence the learning outcomes. Methods were proposed by educational data miners to detect students' affects based on their interactions with the online learning systems (e.g., Baker et al., 2012). By identifying the affects of each student during the learning process, such as boredom, disengagement, confusion, and frustration, educators can provide targeted interventions accordingly to improve learning outcomes. Students can also vary in their preferred mode of instructions. Felder and Silverman (1988) developed the Index of Learning Styles survey, which measured learners' characteristics on the Sensing/Intuiting, Visual/Verbal, Active/Reflective, and Sequential/Global dimensions. A student's learning style can provide indications of possible strengths and difficulties in the learning process.

The increased popularity of computer-based testing has enabled researchers to collect various types of process data, including test takers' reaction time to assessment items, also known as response times. In the field of Psychometrics, extensive research has been conducted on the joint modeling of response accuracy and response times (e.g., Thissen, 1983; van der Linden, 2006, 2007; Fox and Marianti, 2016). Findings from these studies demonstrated that incorporating the additional information from response times, in addition to response accuracy, can improve the estimation accuracy of item parameters and individuals' latent traits or latent classes, further our understanding of individuals' test-taking behavior and the test items' characteristics, and help differentiate learners using different test-taking strategies (e.g., Meyer, 2010; Wang and Xu, 2015). Most recently, response times have been used to measure students' improvements in skill mastery over time. An example is the work from Wang et al. (2018c), in which response times, together with response accuracy, were incorporated into a higher-order hidden Markov model framework (Wang et al., 2018b) to provide information about learners' mastery of the assessed skills, as well as their fluency of applying the mastered skills.

Wang et al. (2018c) assumed that all learners were engaged in the learning process, that is, they devoted their attention to the learning interventions and answered the assessment questions as correctly as possible. However, as mentioned in the very beginning, learners may have different learning styles. Assuming all learners to have the same learning style may under- or over-predict their learning outcomes. This current study aims to address this limitation with a mixture learning model with response times and response accuracy that can account for the presence of heterogeneities in learning styles among learners.

Response times have shown great potentials in identifying students' learning styles, especially student engagement. As an example, Henrie et al. (2015) provided a comprehensive review of methods for measuring student engagement in technology-based learning environments in the literature, and the time spent on homework, web pages, readings, et cetera were commonly used as an indicator of student engagement. Response times were also used by educational data miners to identify disengaged learners Beck (2004). A statistical approach to identify unobserved subpopulations in the data is by using mixture models. Mixture models have been widely used in psychometrics research, for example, addressing some practical issues in testing, such as identifying rapid-guessing or aberrant behaviors among test-takers (e.g., Wang and Xu, 2015), detecting compromised test items (e.g., McLeod et al., 2003), and modeling test-taking speed in time-constrained testing scenarios (e.g., Bolt et al., 2002). A lot of previous research considered the fit of mixture models to response and response time data collected from educational assessments. For example, Wise and DeMars (2006) proposed an effort-moderated IRT model, under which whether or not the response time of an examinee on a test item exceeds an item-specific threshold is used to infer if the subject has demonstrated efforts on the item, and Wang and Xu (2015) used different underlying response and response time distributions for item responses in different test-taking modes (e.g., solution, pre-knowledge, or rapid-guessing). However, modeling heterogeneity in learning behavior is more challenging than modeling that in testing behavior, as one needs to consider different measurement models as well as the transition models that describe the change of latent constructs over time. The proposed model, which will be described in details in the following section, is more closely related to the literature about Mixture Hidden Markov Models (HMMs). Langeheine and Van de Pol (1990) and Van de Pol and Langeheine (1990) proposed the mixed Markov latent class model, which, in its most general form, is the mixture of several first order hidden Markov models. It assumeed that different subpopulations differed in their initial state distributions, transition probabilities, and the response distributions under a HMM. Vermunt et al. (2008) further extended the mixed Markov latent class model to incorporate time-invariant or time-dependent covariates for each subject at each time point.

The mixture learning model proposed in this study adopts a similar framework for modeling the learners' behaviors in a learning process as that in the mixture HMMs. However, instead of assuming subpopulations of learners throughout the entire learning process, we assume that at each point in time, a learner can be in different learning modes. Furthermore, in addition to the item response data, learners' response times are also used in the measurement model, to measure both the change of learners' latent speed over time and any change in their engagement with the learning process.

The rest of the paper is organized as follows. A motivating example is first presented to demonstrate the utility of response times and response accuracy in the detection of heterogeneous learning behaviors in a computer-based learning program. This is followed by the presentation of the proposed mixture learning model and a Bayesian estimation procedure. We then present the results from fitting the proposed mixture model to the data described in the motivating example. A simulation study is presented to verify the accuracy of proposed estimation algorithm under different conditions and to validate the results from the real data analysis. We further discuss our findings and limitations of this study and propose future research directions in the last section.

## 2. A Motivating Example

**Figure 1:**
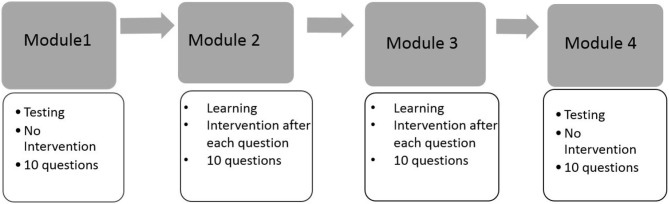
The design of the spatial rotation learning program.

This motivating example is presented to illustrate the necessity of using both response times and response accuracy to differentiate learners' behaviors in a learning environment. We start with presenting the results from an exploratory analysis on a data set collected through a spatial rotation learning program (Wang et al., 2018a). This learning program was developed on the basis of a pilot learning program in Wang et al. (2018b) to train four fine-grained mental rotation skills, namely (1) *x*90: 90° rotation along the x-axis; (2) *y*90: 90° y-axis; (3)*x*180: 180° x-axis; and (4) *y*180: 180° y-axis. Test questions in this new learning program were developed based on the ones in Wang et al. (2018b), and these four distinct yet related skills were identified as the set of measured skills by several previous studies (e.g., Maeda et al., 2013; Culpepper, 2015; Wang et al., 2018b). The structure of the learning program is summarized by the flow chart in Figure 1. Specifically, the learning program started with a testing module, followed by two consecutive learning modules, and finally ended with a testing module. Each module was composed of 10 different questions that were selected based on various criteria, including item characteristics and how well they assessed or improved the four skills. The main purpose of the two testing modules was to measure accurately the four binary spatial skills at a given point in time, while the two learning modules aimed to improve test-takers' mental rotation skills. Learning interventions were provided only in learning modules, in which participants were provided with learning materials after completing each question. A total of 585 undergraduate students with diverse backgrounds participated in the experiment. Written informed consent was obtained from the participants of this study. These students either received a course credit or a stipend through their participation. For students who received a stipend, their total amount of payment was proportional to the number of correct responses they provided in the experiment. Different incentive strategies may also trigger different learning patterns.

**Figure 2:**
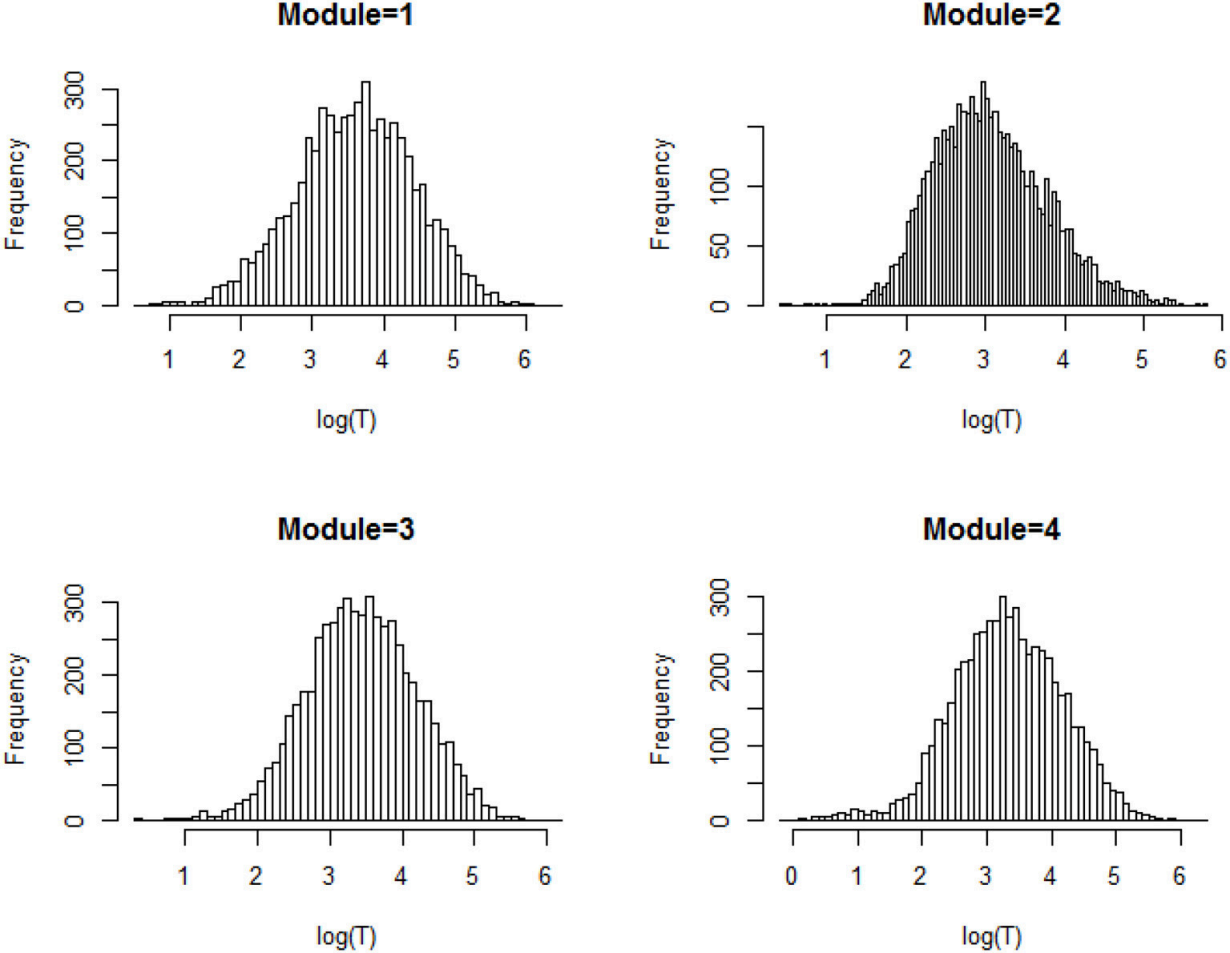
Histogram of the log response time for all person-item combination across four modules.

**Figure 3:**
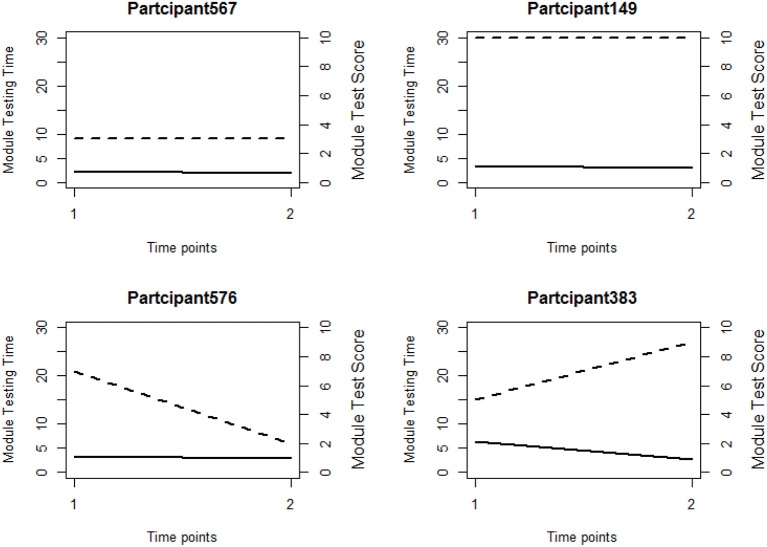
Line plots of four participants' testing time and module scores in module 1 and module 4. The dashed line and solid line represent module score and testing time respectively. Time point 1 represents module 1 and Time point 2 represents module 4.

**Figure 4:**
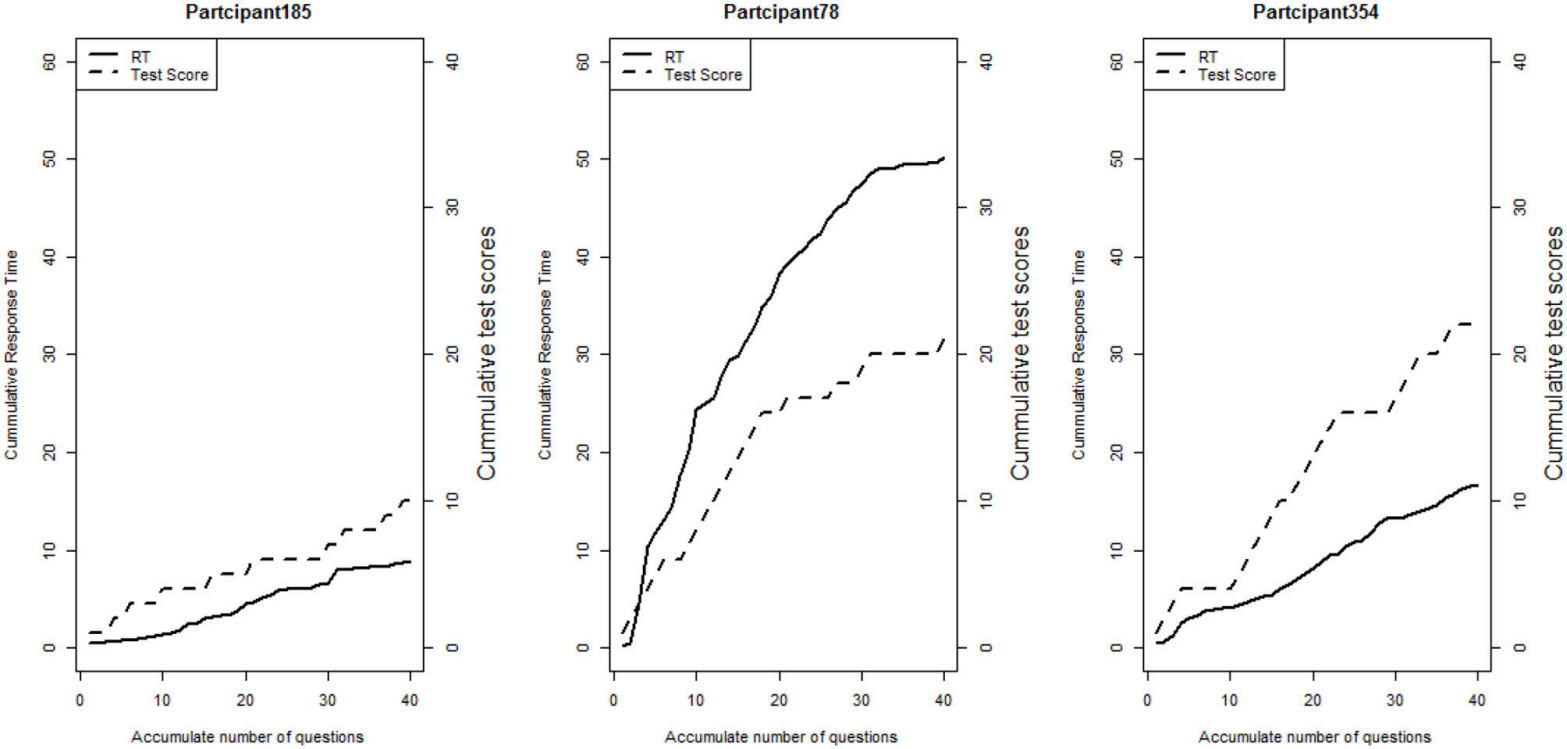
Line plots of three participants' cumulative total scores and total response times at the end of each item.

We first explore the data by plotting the log response times of all person-item combinations across four modules in Figure 2. It is observed that the response time distributions in modules 1, 2, and 4, especially module 4, have a bimodal structure: the first mode appeared within a short time period, while the second appeared at a later time. The previous studies that had similar observations in a testing environment concluded these two modes represent rapid-guessing and solution behaviors, and this is the evidence for a mixture of two populations with different response behaviors (e.g., Van der Linden and Guo, 2008; Wang et al., 2016). However, in a learning environment, the behavior of fast test-taking does not directly imply random guessing, as there is a confounding factor that the speed, especially in module 4, may be due to the improvement of cognitive skills after receiving learning interventions. To see this, we further identified the faster participants in module 4 and explored their module 4 test scores as well as their testing time and module 1 score. The reason to choose module 1 and 4 is because these two testing modules had similar item characteristics and can be regarded as parallel, thus we can compare the change in response accuracy and response times without worrying about the form effect. Figure 3 documented the results from four participants. From there we can see that first, Participant 567 and Participant 145 almost had the same speed in modules 1 and 4. However, the former may represent a person with random guessing as he/she had low response accuracy in both modules, and the latter may represent one who mastered or was fluent in the four skills so that he/she can responded quickly while maintaining high accuracy (achieved a full score in each module). The behavior from Participant 576 may indicate this student had a solution behavior in module 1 but switched to random guessing in model 4. The response speed and response accuracy from Participant 383 both increased, and the increased speed may be due to the improvement of the spatial skills. Lastly, participants may switch engagement mode during the learning process. Figure 4 further documents the examples of learning behaviors of three participants in this experiment. Across all four modules, participant 185 (left) responded to the questions with high speed and low accuracy, indicating he/she was not engaged during the whole experiment. Participant 78 (middle) seemed to be engaged in learning during the first 2 modules, however, his/her response accuracy sharply decreased in module 4 together with the total response time reaching a plateau, indicating he/she started to lose motivation in the last module. Participant 354 (right) presents another pattern, where he/she might not be engaged in the first module, but then switched to be engaged in the following modules. All these findings illustrate the necessity to use response times and response accuracy together to detect different learning behaviors.

## 3. Mixture Learning Model With Response Times and Response Accuracy

### 3.1. Model Formulation

We introduce the mixture modeling framework using the computer-based learning environment presented in section 2 as an example. It is assumed that *N* learners are trained to learn *K* skills at *T* time points, and that they are assessed with items developed under the Diagnostic Classification Model framework (DCM; also known as Cognitive Diagnosis Model). At time point *t*, *J*_*t*_ questions are administered, and the skills measured by each question are documented through a ***Q*_*t*_**-matrix, with the *j, k*th element indexed by *q*_*jkt*_, which equals 1 when item *j* requires attribute *k* and 0 otherwise. Let ${\text{X}}_{it}={\left(X_{i,1,t},\ldots ,X_{i,J_{t},t}\right)}^{\prime }$ denote responses to the *J*_*t*_ questions from learner *i* at time *t*. *X*_*i, j, t*_ takes a value of 1 or 0 depending on whether the response is correct or incorrect. The reaction times, or latencies, for these questions are denoted by ${\text{L}}_{i,t}={\left(L_{i,1,t},\ldots ,L_{i,J_{t},t}\right)}^{\prime }$. For learner *i*, the latent skill profile at time *t* is denoted by ${\text{α}}_{i,t}={\left[\alpha_{i,1,t},\ldots ,\alpha_{i,K,t}\right]}^{\prime }$, with α_*i, k, t*_ = 1 indicating mastery of a skill *k* and α_*i, k, t*_ = 0 indicating non-mastery. Let *D*_*i, t*_ be a binary variable that denotes the learning mode of learner *i* at time point *t*, with *D*_*i, t*_ = 0 for an engaged mode and *D*_*i, t*_ = 1 for a disengaged mode. In this study, we index the time points in the learning process at the module level, that is, each model is regarded as a time point, and a learner is assumed to have the same learning mode and attribute pattern across all items that are administrated at the same time point. We impose this assumption for the consideration of model simplicity, and a generalization of this assumption to item-level time indexing is provided in the discussion section as a future direction. Given the learner's engagement mode at a given time point *D*_*i, t*_, the proposed mixture learning model considers the between-mode differences of the learners on the following three sub-models, namely (1) a transition model that captures the change of latent profile between two adjacent time points, (2) a measurement model that describes the distribution of item responses to the assessment questions at a given time point, and (3) a response time model that outlines the distribution of reaction times at a given time point. As the learner is assumed to have only two modes at a given time point, we will address the above three types of models based on whether the learner is in an engaged learning mode or a disengaged learning mode.

First, a learner in an engaged learning mode (*D*_*i, t*_ = 0) is assumed to employ relevant skills to respond to the assessment questions as accurately as possible. In this case, a reasonable DCM can be chosen as the measurement model. For example, if the deterministic input, noisy-“and”-gate (DINA; e.g., Macready and Dayton, 1977; Junker and Sijtsma, 2001) model is chosen, then the probability of a correct response on item *j* by learner *i* at time *t* is given by

(1) $$\begin{array}{r} P\left(X_{i,j,t}=1|{\text{α}}_{i},s_{j},g_{j},{\text{q}}_{j}\right)={\left(1-s_{j}\right)}^{\eta_{ij}}g_{j}^{1-\eta_{i,j,t}}, \end{array}$$

where $\eta_{i,j,t}=\prod_{k=1}^{K}\alpha_{i,k,t}^{q_{j,k,t}}$ is the ideal response, indicating whether learner *i* possesses all required skills to answer item *j* correctly, and *s*_*j*_ and *g*_*j*_ are the slipping and guessing parameters of item *j*. Essentially, the DINA model describes the case where a learner needs to master all requisite skills of an item to be able to answer the item correctly with high probability (1−*s*_*j*_). Missing any of the item's requisite skills would result in a probability of a correct response of *g*_*j*_ instead. We note that while the DINA model is chosen in the present study, other DCMs can be chosen based on the specific assessment items in hand. This includes, for example, the deterministic input, noisy-“or”-gate model (Templin and Henson, 2006), the reduced reparameterized unified model (DiBello et al., 1995; Hartz, 2002; Roussos et al., 2007), and other general models, such as the log-linear cognitive diagnosis model (Henson et al., 2009), the general diagnostic model (von Davier, 2008), and the generalized-DINA model (de la Torre, 2011).

When a learner engages in solution behavior on an assessment item, we adopt the dynamic response time model proposed by Wang et al. (2018c) to describe the distribution of the reaction time to this item. Specifically, *L*_*i, j, t*_ is assumed to follow a log-normal distribution,

(2) $$\begin{array}{r} \log\left(L_{i,j,t}\right)\sim N\left(\gamma_{j}-\left(\tau_{i}+\phi *G_{i,j,t}\right),\frac{1}{a_{j}^{2}}\right). \end{array}$$

where *τ*_*i*_ is the initial latent speed of learner *i*, γ_*j*_ is the time-intensity parameter of item *j*, capturing the overall amount of time the item requires, and *a*_*j*_ is the time-discrimination parameter of item *j*, which captures variance of log-response times at a given *τ*_*i*_ and γ_*j*_. *G*_*i, j, t*_ is a covariate defined according to the latent skill profile ***α***_*i, t*_, and *ϕ* is the parameter that characterizes the change of the latent speed due to *G*_*i, j, t*_. The key part of such a dynamic response time model is the covariate *G*_*i, j, t*_, which captures the change in speed of the subject over time as a function of the attribute trajectory of subject *i*, and here we use the indicator function for *G* proposed in Wang et al. (2018c), namely

(3) $$G_{i,j,t}=\{\begin{array}{ll} 1, & \text{if }\alpha_{i,t}≽{\text{ q}}_{j},\\ 0, & \text{otherwise.} \end{array}$$

With *G*_*i, j, t*_ defined this way, a learner can take one of two speed statuses on each item: Depending on whether all the required skills of item *j* are mastered time *t*, his or her speed on the item is either *τ*_*i*_ or *τ*_*i*_+*ϕ*. In terms of the transition probability, we make the assumption that a learner in the engaged mode also has high a engagement level in the learning process and thus may improve in skill mastery over time. In the engaged learning mode, the learner's transitions of attribute pattern from that time point to the next is hence modeled using a simplified version of the higher order hidden Markov DCM (HO-HM DCM) proposed by Wang et al. (2018b), specifically, the logit of the probability of transitioning from non-mastery to mastery on skill *k* at time *t*+1 is given by

(4) $$\begin{array}{r} \text{logit}\left[P\left(\alpha_{i,k,t+1}=1|\alpha_{i,k,t}=0,{\text{α}}_{i,t}\right)\right]=\lambda_{0}+\lambda_{1}\theta_{i}+\lambda_{2}\underset{\forall k^{\prime }\neq k}{\sum }\alpha_{i,k^{\prime },t}. \end{array}$$

In this model, *θ*_*i*_ denotes the overall, time-invariant learning ability of learner *i*. The term $\underset{\forall k^{\prime }\neq k}{\sum }a_{i,k^{\prime },t}$ represents how many attributes learner *i* has already acquired other than attribute *k* at time *t*. By using a higher order logistic model for the transition probabilities in the hidden Markov model, the effect of different factors on the probability of learning a skill can hence be examined. A monotonicity assumption is also imposed in the current study, where the probability of forgetting a learned skill, *P*(α_*i, k, t*+1_ = 0∣α_*i, k, t*_ = 1,α_*i, t*_), is 0.

On the other hand, if a learner is in a disengaged learning mode at time *t*, with *D*_*i, t*_ = 1, we assume this learner takes the rapid-guessing strategy on assessment items and shows low engagement in the learning process. We model their rapid-guessing strategy using similar methods as that in Wang and Xu (2015), where the probability of correctly responding to item *j* is equal to a parameter g^*^∈(0, 1) across all items, and the distribution of response times under the rapid-guessing strategy is also assumed to be the same across items, specifically,

(5) $$\begin{array}{r} \log\left(L_{i,j,t}\right)|D_{i,t}=1\sim N\left(\mu_{1},\sigma_{1}^{2}\right), \end{array}$$

where *μ*_1_ and $\sigma_{1}^{2}$ are the mean and variance of the log-response times in the disengaged mode. The disengagement in the learning process is reflected in the transition probabilities from the current stage to the next. In other words, if a learner *i* is in the disengaged mode at time *t*, his or her attribute pattern at time *t*+1 is assumed to be unchanged from α_*i, t*_. As a summary, Table 1 presents the learning, response, and response time models for the learners under two different learning modes.

**Table 1:** Components of the mixture learning model under different engagement modes.

**Learning mode**	**Engaged (*D*_*i, t*_ = 0)**	**Disengaged (*D*_*i, t*_ = 1)**
*P*(α_*i, t*+1_∣α_*i, t*_) =	@l@logit[*P*(α_*i, k, t*+1_ = 1∣α_*i, k, t*_ = 0, **α**_*i, t*_)] =	$I\left(\alpha_{i,t+1}=\alpha_{i,t}\right)$
	$\lambda_{0}+\lambda_{1}\theta_{i}+\lambda_{2}\underset{\forall k^{\prime }\neq k}{\sum }\alpha_{i,k^{\prime },t}$	
*P*(*X*_*i, j, t*_ = 1) =	${\left(1-s_{j}\right)}^{\prod_{k=1}^{K}\alpha_{i,t,k}^{q_{j,k}}}g_{j}^{1-\prod_{k=1}^{K}\alpha_{i,t,k}^{q_{j,k}}}$	g^*^
log(*L*_*i, j, t*_)~	$N\left(\gamma_{j}-\left(\tau_{i}+\phi *G_{i,j,t}\right),\frac{1}{a_{j}^{2}}\right)$	$N\left(\mu_{1},\sigma_{1}^{2}\right)$

### 3.2. Bayesian Estimation

The proposed mixture learning model with engaged and disengaged modes is fitted under a Bayesian framework. We first outline the prior for each parameter in this modeling framework. Recall that *D*_*i, t*_ denotes the membership of learner *i* at time *t* in terms of whether one is disengaged, where *D*_*i, t*_ = 1 if learner *i* is disengaged at time *t*, and *D*_*i, t*_ = 0 otherwise. We assume that

(6) $$\begin{array}{r} D_{i,t}\sim \text{Bernoulli}\left(\omega \right), \end{array}$$

where *ω* is the probability an arbitrary learner belongs to the disengaged group, and the prior distribution of *ω* is

(7) $$\begin{array}{r} \omega \sim \text{Beta}\left(1,1\right). \end{array}$$

The initial attribute pattern of learner *i* is assumed to be a multinomial sample from all *C* = 2^*K*^ possible classes, with

(8) $$\begin{array}{r} P\left(\alpha_{i,1}=\alpha_{c}\right)=\overset{C}{\underset{c=1}{\prod }}\pi_{c}^{I\left(\alpha_{i,1}=\alpha_{c}\right)}, \end{array}$$

where a Dirichlet prior distribution for the initial probabilities of each attribute pattern is used,

(9) $$\pi =[\pi_{1},\ldots ,\pi_{C}]\sim \text{Dirichlet}(1,\ldots ,1).$$

At time *t*∈{1, …, *T*−1}, if a learner is in the engaged learning mode with *D*_*i, t*_ = 0, his or her attribute pattern at the next time point, α_*i, t*+1_, conditioning on the attribute pattern at time *t* is modeled using the HO-HM DCM in Equation (4). Similar to Wang et al. (2018c), we used the following prior probabilities for the learning model parameters:

(10) $$\begin{array}{ll} \lambda_{0} & \sim \text{Normal}\left(0,1\right),\;\lambda_{1}\sim \text{Log-normal}\left(0.5,1\right),\\ \lambda_{2} & \sim \text{Log-normal}\left(-0.5,0.6^{2}\right). \end{array}$$

If the learner is disengaged at time *t* with *D*_*i, t*_ = 1, α_*i, t*+1_ is equal to α_*i, t*_ with probability 1.

The responses of a learner under the engaged mode are assumed to follow the DINA model in Equation (1). A Beta prior was used for the slipping and guessing parameters of all the items, in other words,

(11) $$\begin{array}{r} p\left(s_{j},g_{j}\right)\propto s_{j}^{a_{s}-1}{\left(1-s_{j}\right)}^{b_{s}-1}g_{j}^{a_{g}-1}{\left(1-g_{j}\right)}^{b_{g}-1}I\left(0\leq g_{j}<1-s_{j}\leq 1\right). \end{array}$$

On the other hand, the response to an item *j* by a learner in the disengaged mode is assumed to be a Bernoulli sample with success probability g^*^, in other words, $P\left(X_{i,j,t}=1|D_{i,t}=1\right)=g^{*},$ where g^*^ is assumed to have a Beta(1, 1) prior distribution.

At each time point *t* = 1, …, *T*, if *D*_*i, t*_ = 0, subject *i*'s response time on each item follow the log-normal distribution in Equation (2). Similar to that in Wang et al. (2018c), we use the following priors for the response time model parameters:

(12) $$\begin{array}{r} \gamma_{j}\sim N\left(0,1\right),\phi \sim N\left(0,1\right),\text{and}a_{j}^{2}\sim \text{Gamma}\left(1,1\right). \end{array}$$

If *D*_*i, t*_ = 1, the reaction time to each item by learner *i* are assumed to follow the log-normal distribution given in Equation (5), with the following priors for the response time model parameters:

(13) $$\begin{array}{r} \mu_{1}\sim N\left(0,1\right),\text{and}\sigma_{1}^{2}\sim \text{Inv-Gamma}\left(1,1\right). \end{array}$$

Lastly, for each learner, his or her latent learning ability *θ*_*i*_ follows a standard normal prior distribution, and his or her initial latent speed *τ*_*i*_ in the engaged mode is assumed to follow a normal distribution with mean 0 and variance $\sigma_{\tau }^{2}$, where the variance, $\sigma_{\tau }^{2}$, has the Inverse-Gamma prior distribution:

(14) $$\begin{array}{r} \sigma_{\tau }^{2}\sim \text{Inv-Gamma}\left(2.5,1\right). \end{array}$$

The conditional distribution for each parameter can be derived based on the specified priors and the likelihood function of the observed data. The details on the full conditional distributions of the model parameters are presented in Appendix I. A Metropolis-Hastings within Gibbs sampler is developed to iteratively update the parameters by sampling from their conditional distributions. For *θ*_*i*_ and for λ, their conditional distributions do not resemble any known families of distributions, and thus, Metropolis-Hastings (MH) steps are used to update these parameters. A special note for the MCMC algorithm is that when *D*_*i, t*_, = 1, or in other words when a learner is disengaged, the proposed model assumes that the attribute pattern at the next time point, α_*i, t*+1_, is the same as α_*i, t*_. In this case, α_*i, t*_ and α_*i, t*+1_ share the same attribute pattern. When updating the α_*i, t*_s sequentially for each learner, instead of sampling each α_*i, t*_ separately, sets of consecutive α_*i*_s with no transitions in between (e.g., α_*i, t*_ and α_*i, t*+1_, if *D*_*i, t*_ = 1) are sampled together, conditioning on the attribute pattern before the last transition, the learner's attribute pattern after the next transition, and the observed responses and response times at all time points where the underlying attribute pattern is the current one. For example, if student *i* is disengaged at time 1 and engaged at time 2, then the proposed model predicts that, by the assumptions of “no transition” under the disengaged learning mode, the student should have the same attribute pattern at times 1 and 2. Thus, the algorithm samples α_*i*, 1_ and α_*i*, 2_ together, conditioning on π, α_*i*, 3_, and the observed item responses and response times at time 2. The detailed description of the MCMC algorithm for parameter estimation is given in Appendix II.

## 4. Analyzing Learning Behaviors in a Spatial Rotation Learning Experiment

In this section, we apply the proposed mixture learning model to analyze the data in the motivating example. To demonstrate the necessity of fitting this complex model, we in addition fitted two relatively simpler models, one is the model in Wang et al. (2018c), which is a joint model for response accuracy and response time without considering the mixture structure, and the other is an independent model that fit the response accuracy with the HOHM DCM (Wang et al., 2018b) and the response time with a static log-normal model. These three models all converged after 20,000 iterations based on the Gelman-Rubin proportional scale reduction factor (PSRF; Gelman et al., 2014), also known as $\hat{R}$. The last 25,000 iterations were thus used to provide estimates for model parameters. We compared these three models based on the joint Deviance Information Criteria (DIC) and posterior predictive checking. First, the joint DIC for the proposed mixture model is 223104.2, which is the smallest among the three models [joint (224690.7) and independent (226364.1)], indicating a better fit of the proposed mixture model compared with the two simpler models. The testing quantities used in the posterior predictive checking are the minima, maxima, and mean of the change score (total score in Module 4 minus that in Module 1) and change response time (testing time in Module 4 minus that in Module 1). The posterior predictive *p*-values for these quantities are documented in Table 2. In general, an extreme *p*-value (close to 0 or 1) implies that the model cannot be expected to capture this aspect of the data. Based on the results in Table 2 we can conclude that the three models had a similar fit in terms of response accuracy. However, the mixture model had the best fit for the response time portion, as the other two models had extreme *p*-values for the three defined testing quantities. All these results demonstrate that the mixture learning model can improve the data-model fit compared with the two simpler models, and it is necessary to use this model to explore students' learning behaviors.

**Table 2:** Posterior predictive *p*-values for three testing quantities.

**Model**		**Change**	**Score**		**Change**	**Time**
	**Min**	**Mean**	**Max**	**Min**	**Mean**	**Max**
Mixture	0.375	0.558	0.313	0.706	0.770	0.744
Joint	0.605	0.511	0.311	0.942	0.941	0.930
Independent	0.572	0.514	0.287	0.941	0.949	0.924

The average proportion of disengaged participants from the mixture learning model was estimated as $\hat{\omega }=0.03$ (*SD* = 0.004), indicating on average, about 3% of participants were disengaged at each time point. The following analysis focuses on interpreting the learning behaviors and outcomes in the disengaged learning group and engaged learning group.

### 4.1. Disengaged Learning Group

**Figure 5:**
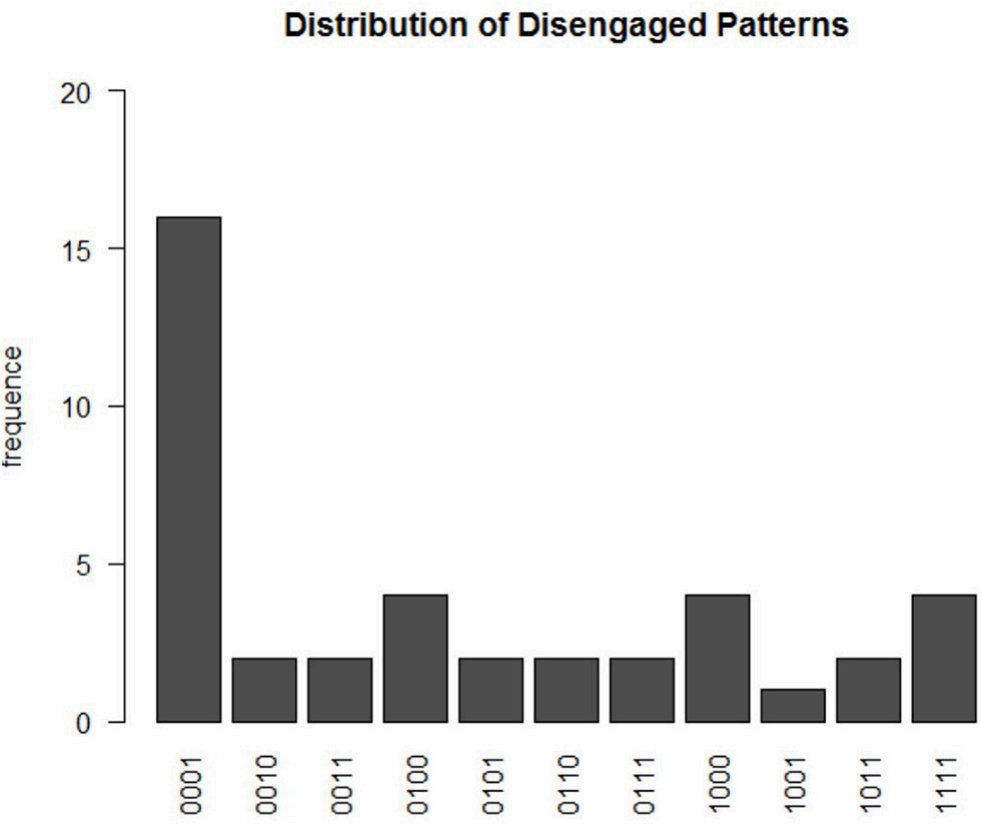
The distribution of disengagement patterns. The x-axis represents the estimated summary pattern of *D*_*i, t*_s at four time points, with 1 indicating disengaged and 0 as engaged.

Based on the estimated ${\hat{D}}_{i,t}$ for each participant *i*, a total of 41 participants were not engaged in at least one of the four time points. There were 11 different disengaged learning patterns, as shown in Figure 5. These patterns can be summarized by four types of disengaged learning behaviors. The first is the behavior that participants began as being engaged in answering questions and learning, but they then became disengaged during the learning process. Among participants with this pattern, a relatively large proportion of them were engaged in learning and testing during the first three modules, but switched to disengaged in the very last module. This could possibly explain the exploratory finding in section 2 that the bimodal structure of the log response time distribution in module 4 is more obvious than that in the other three modules. The second type of behavior can be characterized by the participants being disengaged at first and then switching to engagement in later modules. The third type of behavior is characterized by constant switching between disengaged and engaged modes during the learning process. The last type of behavior is complete disengagement throughout the four modules. These different disengaged behaviors may provide feedback on the learning program design. For example, for the participants who were not engaged in the last module, about 70% of them were estimated to have mastered all four skills after the third module. In the last module, participants may become attuned to the nature of the test or bored, which leads to disengagement. This indicates that varieties in testing questions could be enhanced to better attract their attention in the learning program. When participants was not engaged in answering questions, they randomly guessed the item correctly with probability ĝ^*^ = 0.503(*SD* = 0.022). Their log response time distribution was estimated to follow a normal distribution with mean ${\hat{\mu }}_{1}=2.528\left(SD=0.069\right)$ and variance ${\hat{\sigma }}_{1}^{2}=1.158\left(SD=0.038\right).$ This translates to an expected response time of about 12.5 s per item when a learner is disengaged.

### 4.2. Engaged Learning Group

The posterior means (EAPs) and standard deviations (SDs) estimated with the MCMC algorithm for the coefficients of the transition model and the speed change rate in the response time model are summarized in Table 3. About 52.7% of the participants were estimated as masters of all four skills at the initial time point. In general, when a participant was in an engaged mode, the transition from non-mastery to mastery of a skill at one time point to the next is significantly and positively related to one's general learning ability $\left({\hat{\lambda }}_{1}=2.757\right)$ and the number of mastered skills $\left({\hat{\lambda }}_{1}=0.286\right)$. The speed change rate is estimated as −0.332, indicating participants on average tended to respond more slowly to questions if they mastered the required skills for a question than when they missed some required skills. However, this estimate is the average across all participants; a generalization is to allow each individual to have a different change rate, which could possibly detect the increased speed due to the change of latent skill.

**Table 3:** The MCMC parameter estimates for the transition model and *ϕ* from the response time model.

**Parameters**	***λ*_0_**	***λ*_1_**	***λ*_2_**	***ϕ***
EAP	−2.214	2.757	0.286	−0.332
SD	0.323	0.781	0.119	0.028

*The estimates are the averages across five chains*.

The MCMC estimates for the item parameters, including the DINA model item parameters and the response time model item parameters, are documented in Table 4. The estimated DINA model item parameters are similar to the findings in Wang et al. (2018c) and Wang et al. (2018b), as these two learning programs share similar test questions. The average of the estimated time intensity parameters is 3.10, indicating participants in the engaged mode spent about 22.2 s answering a test question.

**Table 4:** The MCMC parameter estimates for item parameters and response time parameters.

**Item**	***s***	***g***	***a***	**γ**
1	0.045 (0.014)	0.811 (0.043)	1.410 (0.044)	2.312 (0.040)
2	0.086 (0.018)	0.737 (0.044)	1.776 (0.056)	2.940 (0.037)
3	0.082 (0.019)	0.699 (0.037)	1.865 (0.059)	3.371 (0.035)
4	0.224 (0.026)	0.635 (0.033)	1.679 (0.054)	3.762 (0.035)
5	0.140 (0.023)	0.484 (0.039)	1.781 (0.055)	3.452 (0.034)
6	0.223 (0.025)	0.570 (0.037)	1.702 (0.053)	3.476 (0.035)
7	0.195 (0.026)	0.355 (0.040)	1.869 (0.058)	3.510 (0.033)
8	0.195 (0.025)	0.530 (0.035)	1.737 (0.055)	3.658 (0.034)
9	0.299 (0.029)	0.379 (0.036)	1.735 (0.058)	3.687 (0.035)
10	0.279 (0.029)	0.378 (0.035)	1.533 (0.047)	3.612 (0.037)
11	0.019 (0.008)	0.876 (0.039)	2.103 (0.067)	2.671 (0.034)
12	0.011 (0.006)	0.943 (0.019)	2.271 (0.074)	2.594 (0.033)
13	0.037 (0.010)	0.842 (0.043)	2.113 (0.067)	2.601 (0.033)
14	0.088 (0.015)	0.843 (0.038)	2.150 (0.071)	2.464 (0.034)
15	0.106 (0.014)	0.855 (0.029)	2.155 (0.071)	2.187 (0.035)
16	0.064 (0.015)	0.585 (0.042)	1.820 (0.057)	3.040 (0.035)
17	0.095 (0.019)	0.498 (0.047)	2.011 (0.066)	3.019 (0.035)
18	0.060 (0.013)	0.783 (0.034)	1.975 (0.062)	2.854 (0.034)
19	0.089 (0.016)	0.658 (0.040)	1.723 (0.053)	3.135 (0.036)
20	0.119 (0.019)	0.613 (0.042)	1.655 (0.051)	3.179 (0.037)
21	0.032 (0.010)	0.798 (0.051)	1.848 (0.058)	2.630 (0.035)
22	0.220 (0.022)	0.317 (0.043)	1.769 (0.055)	3.292 (0.035)
23	0.329 (0.025)	0.405 (0.045)	1.947 (0.060)	2.979 (0.034)
24	0.135 (0.019)	0.429 (0.065)	1.500 (0.046)	3.173 (0.040)
25	0.257 (0.024)	0.421 (0.049)	2.099 (0.065)	2.904 (0.034)
26	0.146 (0.020)	0.261 (0.042)	1.817 (0.056)	3.333 (0.035)
27	0.215 (0.023)	0.392 (0.041)	1.732 (0.053)	3.509 (0.036)
28	0.361 (0.026)	0.370 (0.040)	1.810 (0.056)	3.395 (0.034)
29	0.483 (0.026)	0.327 (0.041)	1.749 (0.054)	3.289 (0.035)
30	0.532 (0.026)	0.273 (0.037)	1.743 (0.055)	3.271 (0.036)
31	0.063 (0.013)	0.756 (0.060)	2.108 (0.070)	2.622 (0.034)
32	0.035 (0.009)	0.825 (0.044)	2.106 (0.071)	2.264 (0.035)
33	0.033 (0.009)	0.892 (0.030)	1.867 (0.059)	2.736 (0.036)
34	0.227 (0.022)	0.458 (0.049)	1.701 (0.053)	3.241 (0.036)
35	0.141 (0.019)	0.537 (0.048)	1.727 (0.054)	3.075 (0.036)
36	0.205 (0.022)	0.520 (0.049)	1.780 (0.057)	3.498 (0.036)
37	0.232 (0.023)	0.345 (0.043)	1.546 (0.049)	3.492 (0.038)
38	0.274 (0.024)	0.366 (0.044)	1.662 (0.052)	3.299 (0.037)
39	0.494 (0.027)	0.171 (0.034)	1.373 (0.042)	3.439 (0.041)
40	0.254 (0.024)	0.285 (0.044)	1.368 (0.043)	3.206 (0.041)

*Standard errors in parentheses*.

## 5. Simulation Study

A simulation study was conducted to achieve three goals. The first was to verify the accuracy of the proposed MCMC algorithm, the second was to provide validation for the real data analysis, and the last was to demonstrate the necessity of modeling the heterogeneity of learning behaviors when they do exist. In order to achieve these goals, the proposed mixture learning model was chosen as the data generation model and the true model parameters were generated according to the estimated parameters from the real data analysis. Two additional factors were considered, one was sample size (*N* = 585, 1, 000, 3, 000) and the other was the overall probability of disengagement (*ω* = 0.03 or 0.10). Under each simulation condition, 50 data sets were simulated, and the proposed model was refitted through the MCMC algorithm. In addition, under each of the two *N* = 385 conditions (*ω* = 0.03 or 0.10), one data set generated from the mixture model was also fitted to the joint learning model of responses and response times under the HO-HM DCM framework proposed by Wang et al. (2018c). This assumes all learners are in the engaged mode across all time points, and the results from this model misspecification scenario can be used to demonstrate the third goal. The estimated parameters were then compared to the ones used to generate the data sets. The details of the simulation procedures and evaluation criteria are presented in the following subsection.

### 5.1. True Parameters

We simulated the attribute trajectories of *N* = 585, 1, 000, or 3, 000 learners on *K* = 4 skills across *T* = 4 time points. Ten assesment items were administred at each time point (*J*_*t*_ = 10). The learners' initial attribute patterns were randomly sampled from the set of all possible attribute profiles ({0, 1}^*K*^), with probabilities of each profile set to be the expected a posteriori (EAP) estimates from the real data analysis. For each learner, their latent learning ability *θ*_*i*_ was randomly sampled from the standard normal distribution, and their latent speed *τ*_*i*_ was randomly generated from a normal distribution with mean 0 and variance $\sigma_{\tau }^{2}$ estimated from the empirical data.

At each time point *t* = 1, …, *T*, the learners were randomly assigned to one of two possible learning modes, namely the engaged learning mode (*D*_*i, t*_ = 0) and the disengaged learning mode (*D*_*i, t*_ = 1). The true probability of *D*_*i, t*_ = 1 was set to either *ω* = 0.03 or *ω* = 0.1, depending on the simulation condition. Then, conditioning on the learner's mode at time *t*, the attribute mastery changes, responses, and response times were simulated with different distributions. More specifically:

1. **Transition**. If at time *t*, learner *i* is in the engaged learning mode (*D*_*i, t*_ = 0), the probability that the learner transitions from non-mastery to mastery on a skill is given by the modified HO-HM DCM in Equation (4). Similar to Wang et al. (2018b), we assumed the monotonicity in the growth of attribute mastery, in other words, a mastered skill will not be forgotten. The true intercept (λ_0_) and slopes (λ_1_, λ_2_) of the learning model were set to the EAP estimates from the empirical data analysis presented in Table 3. If learner *i* is disengaged at time *t* with *D*_*i, t*_ = 1, the learner's attribute pattern at the next time point, α_*i, t*+1_, was set to be the same as the current one, α_*i, t*_.
2. **Response**. When a learner is in the engaged learning mode at time *t* (*D*_*i, t*_ = 0), the learner is assumed to engage in the solution behavior, and the responses were simulated under the DINA model in Equation (1). The estimated slipping and guessing probabilities from the empirical data were used as the true parameters of the 40 items (Table 4). On the other hand, if the learner is disengaged at time *t* with *D*_*i, t*_ = 1, a rapid-guessing strategy is assumed and the learner's responses are generated from Bernoulli(g^*^). Similar to the other parameters, we set g^*^ equal to the EAP estimate from the real data analysis, which is 0.503.
3. **Response Times**. We assumed that when a learner is in the engaged learning mode, the observed response times follow the log-normal model in Equation (2), with $G_{i,j,t}=I\left(\alpha_{i,t}≽{\text{q}}_{j}\right)$, which takes the value 1 if learner *i* has mastered all requisite skills for item *j* by time *t* and 0 otherwise. For each assessment item, the empirically estimated time intensity parameter γ_*j*_ and time discrimination parameter *a*_*j*_ in Table 4 were used as the true parameters in the simulation study and, similarly, the true value of the slope in front of the covariate *G*_*i, j, t*_, *ϕ* was set equal to the EAP obtained from the real data, which is –0.332. If *D*_*i, t*_ = 1. In other words, learner *i* is disengaged at time *t*, the observed reaction time to any item at that time point was simulated from log-normal(*μ*_1_, σ_1_), again, the EAPs of *μ*_1_ and σ_1_ estimated from the real data were used as the true parameters.

### 5.2. Parameter Estimation

To start the MCMC, we first generated initial values of all the model parameters, and each of them was sequentially updated given the others from the conditional distributions in the Appendix. Specifically, the initial fixed parameters were generated as follows:

$$\begin{array}{l} \lambda_{0}\sim N(0,1),\;\lambda_{1}\sim U(0,1),\;\lambda_{2}\sim U(0,1),\\ \pi \sim \text{Dirichlet}(1),\;\phi \sim U(0,1),\;\omega \sim U(0,0.2),\\ g^{*}\sim U(0,0.5),\;s_{j}\sim U(0,0.3),\;g_{j}\sim U(0,0.3),\\ \mu_{1}\sim N(2,1),\;\sigma_{1}\sim U(0,1),\;\gamma_{j}\sim N(3.45,0.5^{2}),\\ a_{j}\sim U(2,4),\;\sigma_{\tau }^{2}\sim \text{Inv-Gamma}(1,1). \end{array}$$

The random parameters, namely ***D, α, *θ**** and ***τ***, were then randomly generated based on the corresponding fixed parameters.

A chain length of 30, 000 iterations was used for the MCMC, with the first 5, 000 as the burn-in that were excluded for the computation of the point estimates of the parameters. From the post burn-in iterations, we calculated the expected a posteriori (EAP) estimates of each of the model parameters by taking the average of the parameter samples. For the discrete model parameters, α and *D*, the final point estimates were dichotomized depending on whether the associated post burn-in average was < or > 0.5.

### 5.3. Evaluation Criteria

The performance of the proposed algorithm is evaluated in terms of two aspects. The first is to evaluate the convergence of the MCMC algorithm. Five separate chains with different starting values were run with chain lengths of 30, 000 iterations under the *N* = 585, *ω* = 0.1 condition, based on one randomly simulated data set. The $\hat{R}$ (Gelman et al., 2014) was calculated for each parameter at different chain lengths, with the first half of the chain as the burn-in, and the progression of the maximum $\hat{R}$ out of all estimated parameters was used to determine an adequate chain length for convergence. The second was to evaluate the ability of the proposed algorithm to accurately recover the true parameters. The following indices were used to evaluate different parameters in the model. Specifically, the recovery of the learners' attribute patterns of at each time point was evaluated using the attribute-wise agreement rate, $AAR=\frac{\overset{N}{\underset{i=1}{\sum }}\overset{K}{\underset{k=1}{\sum }}I\left(\alpha_{ikt}={\hat{\alpha }}_{ikt}\right)}{N\times K}$, and the pattern-wise agreement rate, $PAR=\frac{\overset{N}{\underset{i=1}{\sum }}I\left(\alpha_{i,t}={\hat{\alpha }}_{i,t}\right)}{N}$, between the true (α) and estimated $\left(\hat{\alpha }\right)$ attribute patterns. Note that the learners who were estimated as not engaged in any of the four time points were excluded from calculating these two indexes, as no information was available to provide estimates for their latent profile at each time point. We further evaluated the recovery of $\phi ,\sigma_{\tau }^{2},\pi ,$ λ, *ω*, *μ*_1_,σ_1_, and g^*^ by comparing the mean and standard deviation of the posterior parameter samples to the true values. The agreement between true and estimated response time model parameters (**a** and γ), learning ability (*θ*), and latent speed (*τ*) was evaluated in terms of the correlation between true and estimated values, and similarly for **a**, ***γ***, ***θ***, **s**, and **g**. Note that for each learner, the data used to update *θ* are the transitions from non-mastery to either non-mastery or mastery at the next time point. Therefore, once a learner becomes a master of all skills, the subsequent αs will not provide additional information on *θ*, and no data on the transitions are available for learners who have mastered all skills at the very beginning. For this reason, the learners whose estimated initial attribute pattern was (1, 1, 1, 1) were excluded from the computation of the correlation between true and estimated learning abilities. The last index is the sensitivity and specificity of the detection of disengagements, that is, the proportion of times that true disengagement is correctly detected, which is defined as $\frac{\overset{N}{\underset{i=1}{\sum }}\overset{T}{\underset{t=1}{\sum }}I\left({\hat{D}}_{i,t}=1,D_{i,t}=1\right)}{\overset{N}{\underset{i=1}{\sum }}\overset{T}{\underset{t=1}{\sum }}I\left(D_{i,t}=1\right)}$), and the proportion of times that true engagement is correctly identified, which is defined as $\frac{\overset{N}{\underset{i=1}{\sum }}\overset{T}{\underset{t=1}{\sum }}I\left({\hat{D}}_{i,t}=0,D_{i,t}=0\right)}{\overset{N}{\underset{i=1}{\sum }}\overset{T}{\underset{t=1}{\sum }}I\left(D_{i,t}=0\right)}$.

### 5.4. Results

#### 5.4.1. Parameter Convergence

**Figure 6:**
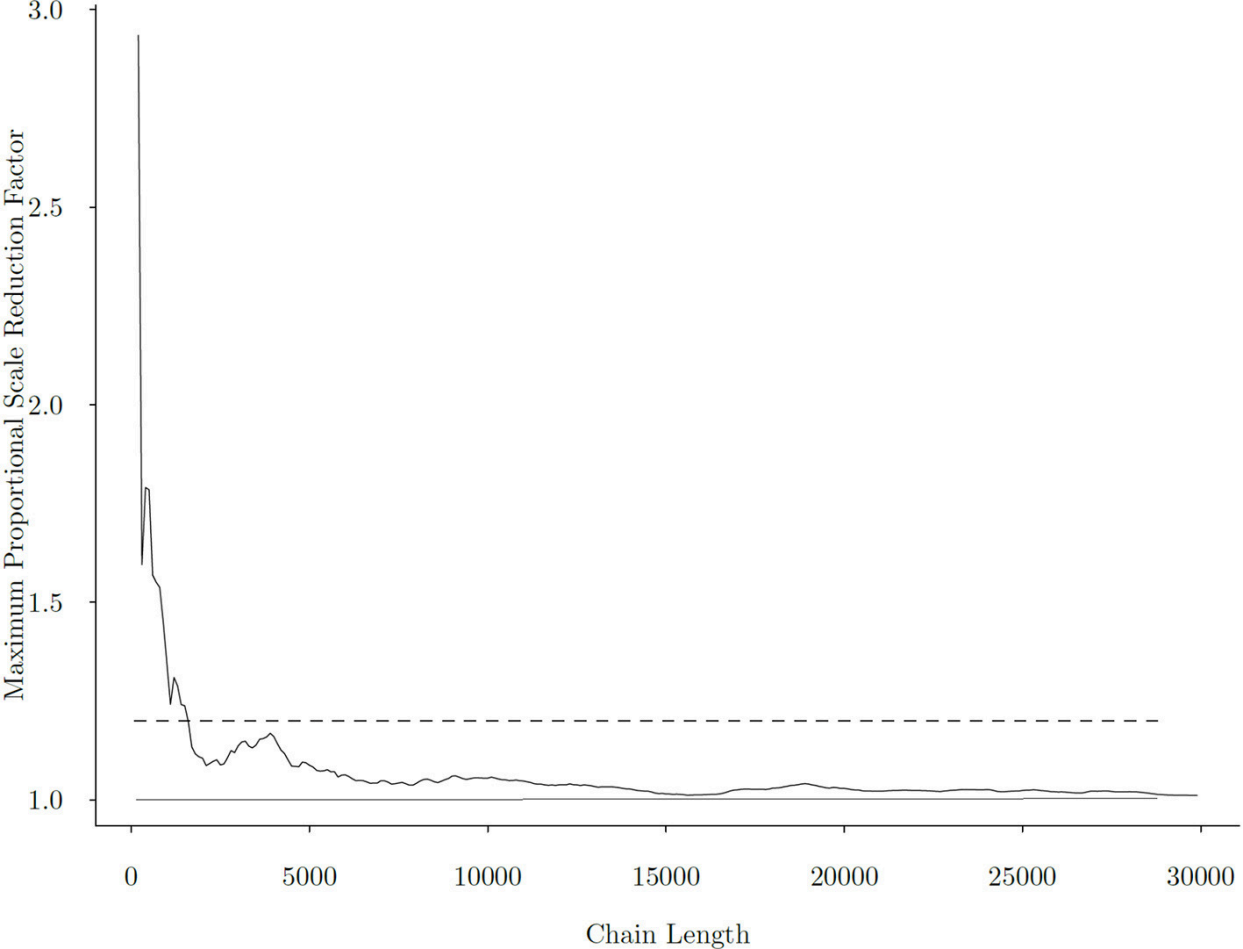
Maximum Gelman-Rubin Proportional Scale Reduction Factor across all parameters with different chain lengths. The *x*−axis is the length of the MCMC chain, and the *y*−axis is the maximum PSRF. The dashed line represents the commonly used threshold of $\hat{R}=1.2$ for parameter convergence, and the solid line corresponds to $\hat{R}=1$, the minimum $\hat{R}$ that can be achieved.

Figure 6 presents the change of the maximum univariate $\hat{R}$ among all model parameters as chain length increases. From the figure, we observe that after approximately 2, 000 iterations, the maximum $\hat{R}$ fell below 1.2, and that at around 5, 000 iterations, $\hat{R}$ has fully stabilized, indicating chain convergence.

#### 5.4.2. Parameter Recovery

Table 5 presents the attribute-wise agreement rates (AARs) and the pattern-wise agreement rates (PARs) between the true and estimated attribute patterns (α) at each time point, under different disengagement rate (*ω*) and sample size (*N*) conditions. Across all conditions and time points in the learning process, the proposed estimation algorithm achieved over 85% accuracy in measuring the presence/absence of attributes for each participant. The estimation accuracy was the lowest for the initial time point (*t* = 1), and it increased as *t* increased, achieving over 90% agreement at *t* = 4. We also observed slightly higher accuracy in the α estimates when sample size was larger and when the probability of disengagement was lower.

**Table 5:** The averaged attribute-wise and pattern-wise agreement rates (AARs and PARs) between the true and estimated α across 50 repetitions under each simulation condition.

**ω**	***N***	**Criteria**	***t* = 1**	***t* = 2**	***t* = 3**	***t* = 4**
0.03	585	AAR	0.872	0.910	0.923	0.923
		PAR	0.683	0.745	0.784	0.793
	1,000	AAR	0.875	0.912	0.926	0.926
		PAR	0.688	0.749	0.789	0.798
	3,000	AAR	0.877	0.913	0.927	0.928
		PAR	0.696	0.752	0.792	0.800
0.10	585	AAR	0.864	0.901	0.916	0.915
		PAR	0.666	0.726	0.769	0.776
	1,000	AAR	0.869	0.903	0.917	0.918
		PAR	0.678	0.732	0.770	0.782
	3,000	AAR	0.872	0.905	0.919	0.919
		PAR	0.688	0.738	0.775	0.784

In Table 6, we present the biases and RMSEs of the fixed parameters in the model and the sensitivity and specificity of the learning mode estimates (*D*_*i, t*_) averaged across 50 replications. Specifically, these fixed parmeters include the transition model's intercept (λ_0_) and slopes (λ_1_, λ_2_), the correct response probability in the disengaged mode (g^*^), the probability of disengagement (*ω*), the mean (*μ*_1_) and standard deviation (σ_1_) of the log response times in the disengaged mode, the coefficient for the increase of latent speed (*ϕ*) for engaged learners, and the variance of latent speed $\left(\sigma_{\tau }^{2}\right)$. Across all conditions, the bias of the estimated fixed parameters, except those associated with the transition model (λ), were relatively small, with small RMSEs. One possible reason for the relatively large bias and RMSE for λ is that with *T* = 4, each learner could be observed on at most 3 transitions, and considering that a large proportion of learners started with mastery of all or most of the skills at the initial time point and that some learners might be disengaged at a selection of time points, the actual number of observations for transitions is usually < 3 per learner. Thus, the amount of data available for estimating the transition model parameters, as well as the *θ*s, is limited. We further observed that larger sample sizes were associated with slightly lower bias and standard error of the parameter estimates. In addition, a higher rate of disengagement (*ω* = 0.1) was associated with larger biases and RMSEs of learning model parameter (λ) and *ϕ* estimates, but smaller biases and RMSEs of g^*^ and *μ*_1_, σ_1_, the parameters associated with the response and response time distributions in the disengaged mode. This trend is expected, as a higher *ω* translates to a larger number of observations associated with disengagement and less observations associated with engagement.

**Table 6:** The bias and RMSE of the fixed parameter estimates under different simulation conditions and the specificity and sensitivity of the *D*_*i, t*_ estimates.

**True *ω***	***N***	**λ_0_ (RMSE)**	**λ_1_ (RMSE)**	**λ_2_ (RMSE)**	g^*^ (RMSE)
0.030	585	0.562 (0.631)	−0.423 (0.775)	0.043 (0.145)	0.001 (0.026)
0.030	1,000	0.430 (0.499)	−0.408 (0.626)	0.037 (0.135)	0.001 (0.022)
0.030	3,000	0.231 (0.307)	−0.204 (0.352)	−0.004 (0.106)	0.003 (0.013)
0.100	585	0.628 (0.691)	−0.556 (0.837)	0.059 (0.153)	−0.000 (0.014)
0.100	1,000	0.479 (0.546)	−0.359 (0.646)	0.037 (0.137)	0.001 (0.011)
0.100	3,000	0.230 (0.313)	−0.225 (0.388)	0.014 (0.119)	0.001 (0.007)
**True** *ω*	*N*	*ω* **(RMSE)**	*μ*_1_ **(RMSE)**	σ_1_ **(RMSE)**	*ϕ*_0_ **(RMSE)**
0.030	585	0.000 (0.005)	−0.000 (0.066)	0.003 (0.042)	−0.017 (0.031)
0.030	1,000	0.000 (0.004)	−0.001 (0.048)	−0.004 (0.033)	−0.010 (0.023)
0.030	3,000	0.000 (0.003)	−0.001 (0.030)	−0.000 (0.022)	−0.003 (0.012)
0.100	585	0.001 (0.008)	−0.000 (0.034)	0.000 (0.023)	−0.023 (0.033)
0.100	1,000	−0.001 (0.007)	−0.001 (0.027)	−0.002 (0.019)	−0.005 (0.022)
0.100	3,000	0.000 (0.004)	−0.003 (0.014)	0.001 (0.010)	−0.005 (0.014)
**True** *ω*	*N*	$\sigma_{\tau }^{2}$ **(RMSE)**	*D***P: Sensitivity**	*D***: Specificity**
0.030	585	0.005 (0.015)	0.952	0.999
0.030	1,000	0.003 (0.010)	0.954	0.999
0.030	3,000	0.001 (0.005)	0.954	0.999
0.100	5,85	0.005 (0.015)	0.967	0.996
0.100	1000	0.002 (0.010)	0.965	0.996
0.100	3,000	0.001 (0.005)	0.967	0.996

Values outside the parenthesis for $\lambda ,g^{*},\omega ,\mu_{1},\sigma_{1},\phi_{0},$ and $\sigma_{\tau }^{2}$ are the biases of the parameter estimates averaged across 50 replications. Values in the parenthesis are the RMSEs of the parameter estimates averaged across 50 replications.

Across several repetitions of the simulation study, the estimated learning mode of each learner at each time point, *D*_*i, t*_, showed high agreement with the true values, with sensitivity over 95% when *ω* = 0.03 and over 96% when *ω* = 0.1, and specificity over 99% across all simulation conditions. This suggests that under the proposed estimation algorithm, whether a learner is disengaged or engaged at a given time point could be detected correctly most of the times based on their response times, responses, and transitions in attribute mastery.

The correlation between true and estimated values of ***θ***, *τ*, **a**, γ, **s**, and **g** are presented in Table 7. For the items' response time model parameters (**a**,γ), the DINA model parameters (**s**, **g**), and the learners' initial latent speeds (*τ*), there was a high agreement between the true and estimated values, with correlations over 96%. For the latent learning abilities of the learners (*θ*), the estimate values demonstrated larger errors with correlations around 0.67 when *ω* = 0.03 and around 0.64 when *ω* = 0.1. Similar to the larger errors in the transition model parameter estimates, we think the larger error in the estimation of *θ* can potentially be attributed to the paucity of data available to update *θ*_*i*_ for each subject.

**Table 7:** Correlations between true and estimated latent learning ability (*θ*) and initial speed (*τ*) of learners, item response time model parameters (**a**,γ), and DINA model item parameters (**s**, **g**).

***ω***	***N***	**ρ_*θ*_**	**ρ_*τ*_**	**ρ_*a*_**	**ρ_γ_**	**ρ_*s*_**	**ρ_*g*_**
0.03	585	0.663	0.968	0.967	0.998	0.989	0.982
	1,000	0.670	0.968	0.982	0.999	0.993	0.989
	3,000	0.669	0.968	0.994	1.000	0.998	0.997
0.10	585	0.643	0.964	0.967	0.998	0.988	0.982
	1,000	0.647	0.964	0.979	0.999	0.993	0.989
	3,000	0.645	0.965	0.993	1.000	0.997	0.996

#### 5.4.3. Consequences of Misspecification

Finally, we briefly summarize the parameter recovery results when the model is misspecified, that is, when the data generating model is the mixture model but the mixture structure is ignored when refitting data. We note that this is a special case of the proposed mixture model with *D*_*i, t*_ = 0 for all *i* and *t*.

Table 8 presents the summary of the parameter recovery results when the model without mixture is fitted to the data generated from the mixture learning model, with different true disengagement probabilities (*ω* = 0.03 or 0.10). In both cases, a sample size of *N* = 585 was used. We present the correlations between the true and estimated *θ*,*τ*, **a**,γ, **s**, and **g**. In addition, we also present the averaged attribute agreement rate (*AAR*) between true and estimated α across the four stages.

**Table 8:** Recovery of the model parameters when the mixture in the data is ignored.

**True *ω***	**ρ_*θ*_**	**ρ_*τ*_**	**ρ_*a*_**	**ρ_γ_**	**ρ_*s*_**	**ρ_*g*_**	**$\bar{AAR}$**
0.03	0.561	0.904	0.926	0.997	0.983	0.958	0.859
0.10	0.426	0.815	0.827	0.993	0.979	0.965	0.802

Compared to when the mixture is explicitly modeled, ignoring the mixture in the data resulted in remarkable decreases in the estimation accuracy of *θ*, *τ*, **a**, and the attribute trajectories of the learners, α. The decrease in estimation accuracy is more salient when the proportion of disengagement is higher. Thus, we conclude that when learner disengagement exists in the learning process, assuming that all learners are engaged could greatly sabotage the model parameter estimates, including the estimates of the learner's skill mastery patterns and latent traits.

In addition to the recovery of the true model parameters under model misspecification, we also compare the model-data fit of the missepcified model and that of the mixture model. As a reminder, these two models were fitted to the response and response times data generated under the mixture condition with *N* = 585 and *ω* = 0.03. On the same data set, the DIC obtained from the mixture learning model and the misspecified model was 223269.1 and 226197.3, respectively. This suggests that when a mixture structure does exist in the observed data, the model without the mixture fits significantly worse than the mixture model.

## 6. Discussion

In this paper, we propose a mixture learning modeling framework which can address the heterogeneity in learning behaviors. A simple model with two possible learning modes, namely the engaged mode and the disengaged mode, motivated by a real data analysis on a computer-based learning program, is provided as an example. Specifically, with this model, learners are assumed to demonstrate different learning and response behaviors under different modes, leading to differences in the distributions of attribute mastery transitions over time, item responses, and response times. A Bayesian estimation procedure is established to estimate the parameters of the mixture learning model. Different learning behaviors were discovered by applying the proposed model to the real data from the spatial rotation learning program. Simulation studies showed that the model parameters could be accurately estimated, the learners' learning mode could be detected with high accuracy, and the Markov chains stabilized within 5, 000 iterations. In addition, the simulation results from the model misspecification scenario suggested the necessity of fitting the proposed mixture learning model instead of a homogeneous learning model when data suggest the existence of a mixed structure of learning modes.

The proposed mixture learning model has the potential to detect learner disengagement in an online learning context. Compared to traditional classroom learning, online learning programs often provide the learners with a significantly more flexible and less controlled environment. Whereas, instructors in traditional classrooms can directly observe the learners' behaviors and their reactions to different interventions, in online learning, the educators do not interact face to face with the learners. This mixture learning model framework provides a way for educators to infer the online learners' learning mode (e.g., engaged or disengaged) and their corresponding latent skills based on the observed responses and reaction times to assessment questions at different time points. This can help the educators to provide different stimuli to different learners through the online learning environment. Furthermore, the proposed model can also help to refine and design individualized learning materials. As demonstrated from the real data analysis, learners may become disengaged at a certain stage of the learning process, and if this can be detected, then different types of learning materials can be delivered so that it does not make the learning tasks boring or transparent. Finally, even though illustrated within a DCM framework, the way to model the engaged and disenaged learning behavior can be generalized to other latent variable models based on specific assessment requirements. For example, if a continuous latent trait is assumed to be measured by the assessment, then a traditional Item Response Theory Model can be used for response accuracy. The latent growth model can be used to describe the change of the continuous latent trait.

Though promising, the proposed mixture learning model has the limitations that it only considers two learning modes and it assumes the learning mode is the same for all items in the same module. These restrictions can all be relaxed in future studies, in which more than two learning modes can be considered to differentiate various types of disengagement or to capture other learning behaviors other than engagement and disengagement, such as a warm-up mode, where students have low familiarity with the learning environment and need some time to adjust before fully engaging. We can also consider the learners' modes and attribute patterns at a finer grain size, such as treating the response to each item as a time point. Another direction is to consider a higher order model that describes the probability that a learner is disengaged at a specific time point, given a set of time dependent or time independent covariates, such as learners' demographic information or other characteristics, the mode of instruction (e.g., video, text, interactive exercise), or the temporal position of the current learning block (e.g., first learning block which may show slow warm-up of the learners, or later learning blocks on which learners may demonstrate fatigue). Lastly, a prior sensitivity analysis needs to be conducted in the future to investigate the sensitivity of the model estimation results to the prior specification.

## Ethics Statement

The experiment related to the spatial rotation learning program was approved by the Institutional Review Board, office of the Vice President for Research at University of Georgia (IRB ID: STUDY00004215). Before the experiment, participants received a consent form that explains the purpose of this research study, the study procedure, Risks and discomforts, benifits, Incentives for participation, Privacy / Confidentiality. They were totally voluntary to participant in this experiment.

## Data Availability Statement

The real data set analyzed in this manuscript is not publicly available, because it is part of an ongoing research project from the research team. Requests to access the dataset should be directed to Dr. Shiyu Wang, swang44@uga.edu.

## Author Contributions

SZ contributed to the development of the methodological framework and the model estimation procedures, the conduction of the simulation studies, and the drafting and revision of the manuscript. SW contributed to providing suggestions on the methodological framework and the model estimation procedures, the conduction of the real data analyses, and the drafting and revision of the manuscript.

### Conflict of Interest Statement

The authors declare that the research was conducted in the absence of any commercial or financial relationships that could be construed as a potential conflict of interest.
