# Supplementary material for: Modeling Learner Heterogeneity: A Mixture Learning Model With Responses and Response Times
Source: Front Psychol. 2018 Dec 5;9:2339. doi: 10.3389/fpsyg.2018.02339 (PMC6290643; doi:10.3389/fpsyg.2018.02339)
Supplement: Supplementary file 1 [file Data_Sheet_1.pdf]

Modeling Learner Heterogeneity: A Mixture Learning Model with Responses and Response Times

Susu Zhang[1] **and Shiyu Wang**[2*]

[1] *Department of Psychology, University of Illinois at Urbana-Champaign, Urbana, IL, USA*
[2] *Quantitative Methodology Program, Department of Educational Psychology, University of Georgia, Athens , GA, USA*

Correspondence*:
Dr. Shiyu Wang
swang44@uga.edu

APPENDIX I: BAYESIAN FULL CONDITIONAL DISTRIBUTION

Bayesian Full Conditional Distribution

Under the Bayesian modeling framework described in section 3.2, the full likelihood of the learners' responses and response times, as well as their speed, learning ability, and attribute patterns and learning modes at each time point conditioning on the fixed model parameters is given by

$$\begin{aligned}
& P(\boldsymbol{X}, \boldsymbol{L}, \boldsymbol{\alpha}, \boldsymbol{\theta}, \boldsymbol{\tau}, \boldsymbol{D} \mid \boldsymbol{\lambda}, \mathrm{s}, \mathbf{g}, g^*, \mathbf{a}, \boldsymbol{\gamma}, \phi, \mu_1, \sigma_1^2, \omega, \boldsymbol{\pi}, \sigma_\tau^2) \\
= & \prod_{i=1}^{N} \Bigg\{ p(\tau_i \mid \sigma_\tau^2) p(\boldsymbol{\alpha}_{i,t} \mid \boldsymbol{\pi}) \times \\
& \prod_{t=1}^{T-1} \Bigg[P(D_{i,t} \mid \omega) P(\boldsymbol{X}_{i,t}, \boldsymbol{L}_{i,t} \mid D_{i,t}, \cdot) P(\boldsymbol{\alpha}_{i,t+1} \mid D_{i,t}, \boldsymbol{\alpha}_{i,t}, \cdot) \Bigg] \times \\
& P(D_{i,T} \mid \omega) P(\boldsymbol{X}_{i,T}, \boldsymbol{L}_{i,T} \mid D_{i,T}, \cdot) \Bigg\}, \qquad (1)
\end{aligned}$$

where

$$\begin{aligned}
& P(\boldsymbol{X}_{i,t}, \boldsymbol{L}_{i,t} \mid D_{i,t}, \cdot) \\
= & \begin{cases} \prod_{j=1}^{J_t} g^{*X_{i,j,t}} (1-g^*)^{1-X_{i,j,t}} f(L_{i,j,t} \mid \mu_1, \sigma_1^2), & \text{if } D_{i,t} = 1 \\ \prod_{j=1}^{J_t} (1-s_j)^{\prod_{k=1}^{K} \alpha_{i,t,k}^{q_{j,k}}} g_j^{1-\prod_{k=1}^{K} \alpha_{i,t,k}^{q_{j,k}}} f(L_{i,j,t} \mid \gamma_j, \tau_i, \phi, \boldsymbol{\alpha}_{i,t}, a_j), & \text{if } D_{i,t} = 0, \end{cases} \qquad (2)
\end{aligned}$$

and

$$P(\boldsymbol{\alpha}_{i,t+1} \mid D_{i,t}, \boldsymbol{\alpha}_{i,t}, \cdot) = \begin{cases} \mathcal{I}(\boldsymbol{\alpha}_{i,t+1} = \boldsymbol{\alpha}_{i,t}), & \text{if } D_{i,t} = 1, \\ \prod_{k=1}^{K} P(\alpha_{i,t+1,k} \mid \boldsymbol{\alpha}_{i,t}, \boldsymbol{\lambda}, \theta_i), & \text{if } D_{i,t} = 0. \end{cases} \qquad (3)$$

We next present the conditional distribution of each model parameter given the other parameters and the observed responses and response times, which can be used to obtain random samples from the posterior distribution with a MH within Gibbs sampling algorithm.

At each time point t and for each subject i, the conditional distribution of $D_{i,t}$ is

$$P(D_{i,t} = 1 \mid \omega, \boldsymbol{X}_{i,t}, \boldsymbol{L}_{i,t}, \boldsymbol{\alpha}_i) = \frac{\tilde{\pi}_{i,t,1}}{\sum_{d=0}^{1} \tilde{\pi}_{i,t,d}}, \tag{4}$$

where $\tilde{\pi}_{i,t,d}$ is given by

$$\tilde{\pi}_{i,t,d} = \begin{cases} P(D_{i,t} = d \mid \omega) P(\boldsymbol{\alpha}_{i,t+1} \mid D_{i,t} = d, \cdot) P(\boldsymbol{X}_{i,t}, \boldsymbol{L}_{i,t} \mid D_{i,t} = d, \cdot), & \text{if } t < T, \\ P(D_{i,t} = d \mid \omega) P(\boldsymbol{X}_{i,t}, \boldsymbol{L}_{i,t} \mid D_{i,t} = d, \cdot), & \text{if } t = T, \end{cases} \tag{5}$$

where $P(\boldsymbol{\alpha}_{i,t+1} \mid D_{i,t} = d, \cdot)$ and $P(\boldsymbol{X}_{i,t}, \boldsymbol{L}_{i,t} \mid D_{i,t} = d, \cdot)$ could be obtained from Equations (2) and (3).

The conditional distribution of the mixture weight, ω, is

$$\omega \mid \boldsymbol{D} \sim \text{Beta}(1 + \sum_{i=1}^{N} \sum_{t=1}^{T} D_{i,t}, 1 + \sum_{i=1}^{N} \sum_{t=1}^{T} (1 - D_{i,t})). \tag{6}$$

For each subject i and each time point t, the conditional probability that the current attribute pattern $\boldsymbol{\alpha}_{i,t}$ equals $\boldsymbol{\alpha}_c \in \{0,1\}^K$ is

$$P(\boldsymbol{\alpha}_{i,t} = \boldsymbol{\alpha}_c) = \frac{\tilde{\pi}_{ict}}{\sum_{c'=1}^{2^K} \tilde{\pi}_{ic't}}, \tag{7}$$

where $\tilde{\pi}_{ict}$ is given by

$$\tilde{\pi}_{ict} = \begin{cases} \pi_c P(\boldsymbol{\alpha}_{i,t+1} \mid \boldsymbol{\alpha}_{i,t}, D_{i,t}) P(\boldsymbol{X}_{i,t} \mid \boldsymbol{\alpha}_{i,t}, D_{i,t}) f(\boldsymbol{L}_{i,t} \mid \boldsymbol{\alpha}_{i,t}, D_{i,t}), & \text{if } t = 1, \\ P(\boldsymbol{\alpha}_{i,t} \mid \boldsymbol{\alpha}_{i,t-1}, D_{i,t-1}) P(\boldsymbol{\alpha}_{i,t+1} \mid \boldsymbol{\alpha}_{i,t}, D_{i,t}) P(\boldsymbol{X}_{i,t} \mid \boldsymbol{\alpha}_{i,t}, D_{i,t}) \\ \times f(\boldsymbol{L}_{i,t} \mid \boldsymbol{\alpha}_{i,t}, D_{i,t}), & \text{if } 1 < t < T, \\ P(\boldsymbol{\alpha}_{i,t} \mid \boldsymbol{\alpha}_{i,t-1}, D_{i,t-1}) P(\boldsymbol{X}_{i,t} \mid \boldsymbol{\alpha}_{i,t}, D_{i,t}) f(\boldsymbol{L}_{i,t} \mid \boldsymbol{\alpha}_{i,t}, D_{i,t}), & \text{if } t = T. \end{cases} \tag{8}$$

For the population proportions of the attribute patterns at time 1, $\boldsymbol{\pi}$, the conditional distribution is

$$\boldsymbol{\pi} \mid \boldsymbol{\alpha}_{1,1} \ldots, \boldsymbol{\alpha}_{N,1} \sim \text{Dirichlet}(1 + \tilde{N}), \tag{9}$$

where $\tilde{N} = [\sum_{i=1}^{N} \mathcal{I}(\boldsymbol{\alpha}_{i,1} = \boldsymbol{\alpha}_1), \ldots, \sum_{i=1}^{N} \mathcal{I}(\boldsymbol{\alpha}_{i,1} = \boldsymbol{\alpha}_{2^K})]$.

For any learner i, the conditional distribution of θ_i is

$$P(\theta_i \mid \boldsymbol{\alpha}_i, \boldsymbol{\lambda}) \propto p(\theta_i)\Big[\prod_{\substack{t<T:\\ D_{i,t}=0}} P(\boldsymbol{\alpha}_{i,t+1} \mid \boldsymbol{\alpha}_{i,t}, \theta_i, \boldsymbol{\lambda})\Big], \tag{10}$$

and the conditional distribution of τ_i is $N(\mu_{\tau_i}, \sigma^2_{\tau_i})$, where

$$\begin{aligned} \mu_{\tau_i} &= -\frac{\sum_{t=1}^{T}\Big\{(1-D_{i,t})\Big\{\sum_{j=1}^{J_t} a_j^2[\log(L_{i,j,t}-\gamma_j+\phi * G_{i,j,t})]\Big\}\Big\}}{\sum_{t=1}^{T}\Big[(1-D_{i,t})\sum_{j=1}^{J_t} a_j^2\Big]+1/\sigma_\tau^2}, \text{ and} \\ \sigma^2_{\tau_i} &= \frac{1}{\sum_{t=1}^{T}\Big[(1-D_{i,t})\sum_{j=1}^{J_t} a_j^2\Big]+1/\sigma_\tau^2}. \end{aligned} \tag{11}$$

And the conditional distribution of the variance of initial latent speed, σ^2_τ, is

$$\sigma^2_\tau \mid \boldsymbol{\tau} \sim \text{Inv-Gamma}(2.5+\frac{N}{2}, 1+\frac{\sum_{i=1}^{N}\tau_i^2}{2}). \tag{12}$$

For the slopes and intercept of the HO-HM DCM, $\boldsymbol{\lambda}$, the conditional distribution is proportional to

$$p(\boldsymbol{\lambda})\prod_{i=1}^{N}\prod_{\substack{t<T-1:\\ D_{i,t}=0}} P(\boldsymbol{\alpha}_{i,t+1} \mid \boldsymbol{\alpha}_{i,t}, \boldsymbol{\lambda}, \theta_i). \tag{13}$$

The conditional distribution of the DINA model s_j, g_j for each item j is given by

$$P(s_j, g_j \mid \boldsymbol{X}_j, \boldsymbol{\alpha}, \boldsymbol{D}) \propto s_j^{\tilde{a}_s-1}(1-s_j)^{\tilde{b}_s-1} g_j^{\tilde{a}_g-1}(1-g_j)^{\tilde{b}_g-1}\mathcal{I}(g_j<1-s_j), \tag{14}$$

with

$$\tilde{a}_s = 1+\sum_{\substack{i:D_{i,t}=0\\ \& X_{i,j}=0}} \eta_{i,j,t}, \qquad \tilde{b}_s = 1+\sum_{\substack{i:D_{i,t}=0\\ \& X_{i,j}=1}} \eta_{i,j,t},$$

$$\tilde{a}_g = 1+\sum_{\substack{i:D_{i,t}=0\\ \& X_{i,j}=1}} (1-\eta_{i,j,t}), \qquad \tilde{b}_g = 1+\sum_{\substack{i:D_{i,t}=0\\ \& X_{i,j}=0}} (1-\eta_{i,j,t}),$$

where $\eta_{i,j,t}$ denotes the ideal response under the DINA model.

The conditional distribution of correct response probability for learners in the disengaged mode, g^*, is

$$g^* \mid \boldsymbol{X}, \boldsymbol{D} \sim \text{Beta}(\tilde{a}_{g^*}, \tilde{b}_{g^*}), \tag{15}$$

where

$$a_{g^*} = 1 + \sum_{i,t:D_{i,t}=1} \sum_{j=1}^{J_t} X_{i,j,t}, \qquad b_{g^*} = 1 + \sum_{i,t:D_{i,t}=1} \sum_{j=1}^{J_t} (1 - X_{i,j,t}).$$

For each item j, the conditional distribution of the time discrimination parameter a_j^2 is

$$a_j^2 \sim \text{Gamma}\Big(1 + \frac{\sum_{i=1}^N (1 - D_{i,t_{ij}})}{2}, 1 + \frac{\sum_{i=1}^N (1 - D_{i,t_{ij}})(\log L_{i,j,t_{ij}} + \tau_i + \phi G_{i,j,t_{ij}} - \gamma_j)^2}{2}\Big), \tag{16}$$

where t_{ij} denotes the time at which item j is given to subject i. And the conditional distribution of the time intensity parameter, γ_j, is

$$\gamma_j \mid \boldsymbol{L}_j, \boldsymbol{D}, a_j, \phi, \boldsymbol{\tau} \sim N(\tilde{\mu}_\gamma, \tilde{\sigma}_\gamma^2), \text{ with} \tag{17}$$

$$\tilde{\sigma}_\gamma^2 = 1/\Big(1 + a_j^2 \sum_{i=1}^N (1 - D_{i,t_{ij}})\Big),$$

$$\tilde{\mu}_\gamma = \tilde{\sigma}_\gamma^2 * \Big\{a_j^2 \sum_{i=1}^N (1 - D_{i,t_{ij}})(\log L_{i,j,t_{ij}} + \tau_i + \phi G_{i,j,t_{ij}})\Big\}.$$

For ϕ, the slope for the covariate describing speed increase over time in the engaged learning mode, the conditional distribution is

$$\phi \mid \boldsymbol{L}, \boldsymbol{\alpha}, \boldsymbol{\tau}, \mathbf{a}, \boldsymbol{\gamma}, \boldsymbol{D} \sim N(\tilde{\mu}_\phi, \tilde{\sigma}_\phi^2), \text{ with} \tag{18}$$

$$\tilde{\sigma}_\phi^2 = 1/\Big(1 + \sum_{i=1}^N \sum_{t=1}^T (1 - D_{i,t_{ij}}) \sum_{j=1}^{J_t} a_j^2 G_{i,j,t_{ij}}^2\Big),$$

$$\tilde{\mu}_\phi = \tilde{\sigma}_\phi^2 * \Big\{\sum_{i=1}^N \sum_{t=1}^T (1 - D_{i,t_{ij}}) \sum_{j=1}^{J_t} \Big[a_j^2(\gamma_j - \tau_i - \log L_{i,j,t_{ij}}) G_{i,j,t_{ij}}\Big]\Big\}.$$

Lastly, the conditional distributions of the mean and standard deviation of log-response times under the disengaged learning mode are as the following:

$$\mu_1 \mid \boldsymbol{L}, \boldsymbol{D}, \sigma_1^2 \sim N(\tilde{\mu}_{\mu_1}, \tilde{\sigma}_{\mu_1}^2), \text{ with} \tag{19}$$

$$\tilde{\sigma}^2_{\mu_1} = 1/\Big(1 + \frac{1}{\sigma_1^2} J_t \sum_{i=1}^{N} \sum_{t=1}^{T} D_{i,t}\Big),$$

$$\tilde{\mu}_{\mu_1} = \tilde{\sigma}^2_{\mu_1} * \Big\{\frac{1}{\sigma_1^2} \sum_{i=1}^{N} \sum_{t=1}^{T} \left[D_{i,t} \sum_{j=1}^{J_t} \log L_{i,j,t} \right] \Big\}. \text{ And}$$

$$\sigma_1^2 \mid \boldsymbol{L}, \boldsymbol{D}, \mu_1 \sim \text{Inv-Gamma}\Big(1 + \frac{J_t \cdot \sum_{i=1}^{N} \sum_{t=1}^{T} D_{i,t}}{2}, 1 + \frac{\sum_{i=1}^{N} \sum_{t=1}^{T} \left[D_{i,t} \sum_{j=1}^{J_t} (\log L_{i,j,t} - \mu_1)^2 \right]}{2}\Big). \tag{20}$$

APPENDIX II: MARKOV CHAIN MONTE CARLO ALGORITHM FOR PARAMETER SAMPLING

Algorithm 1 MCMC algorithm for mixture learning model parameter sampling.

Inputs:

Observed responses, $\boldsymbol{X}$;
Response times, $\boldsymbol{L}$;
Q-matrices, $\mathbf{Q}$;
Standard deviation of proposal distribution of θ, σ_θ^*
band-widths of proposal distribution for $\boldsymbol{\lambda}$, $\boldsymbol{\delta}^*$;
chain length, R.

Initialization:

Sample transition model parameters, $\lambda_0^{[0]} \sim N(0,1)$, and $\lambda_1^{[0]}, \lambda_2^{[0]} \sim U(0,1)$;
Sample variance of initial speed, $\sigma_\tau^{2[0]} \sim U(1, 1.5)$;
For each learner i, sample $\theta_i^{[0]} \sim N(0,1)$, $\tau_i^{[0]} \sim N(0, \sigma_\tau^{2[0]})$;
Sample $\boldsymbol{\pi}^{[0]} \sim \text{Dirichlet}(1, \ldots, 1)$;
For each learner i, sample $\boldsymbol{\alpha}_{i,1}^{[0]} \sim \text{Multinomial}(\boldsymbol{\pi}^{[0]})$;
Sample the disengagement probability, $\omega^{[0]} \sim U(0, .2)$;
For $i = 1, \ldots, N, t = 1, \ldots, T$, set $D_{i,t}^{[0]} = 0$;
For $t = 2, \ldots, T, i = 1, \ldots, N$, simulate $\boldsymbol{\alpha}_{i,t}^{[0]}$ based on $D_{i,t-1}^{[0]}, \boldsymbol{\alpha}_{i,t-1}^{[0]}, \theta_i^{[0]}$, and $\boldsymbol{\lambda}^{[0]}$;
For each item j, sample DINA parameters $s_j^{[0]}, g_j^{[0]} \sim U(0, .3)$, and the RT parameters $a_j^{[0]} \sim U(2,4)$, $\gamma_j^{[0]} \sim N(3.45, .5^2)$;
Sample $\phi^{[0]} \sim U(0,1)$;
Sample the correct response probability under the disengaged mode, $g^{*[0]} \sim U(0, .5)$;
Sample mean and variance for log-response time under the disengaged mode, $\mu_1^{[0]} \sim N(2,1)$, $\sigma_1^{2[0]} \sim U(0,1)$;

For $r = 1, \ldots, R-1$**, do:**

For $i = 1, \ldots, N, t = 1, \ldots, T$, sample $D_{i,t}^{[r+1]}$ based on Equation (5), given $\boldsymbol{\alpha}_{i,t}^{[r]}, \boldsymbol{\alpha}_{i,t+1}^{[r]}, \theta_i^{[r]}, \boldsymbol{\lambda}^{[r]}, \boldsymbol{X}_{i,t}^{[r]}$, and $\boldsymbol{L}_{i,t}^{[r]}$;
For each i, starting from $t = 1$, based on $\boldsymbol{D}_i^{[r]}$, determine the last time point t^* until which $\boldsymbol{\alpha}_{i,t^*}$ remains unchanged from $\boldsymbol{\alpha}_{i,t}$ according to the model assumption on the transition probability in the disengaged mode, sample $\boldsymbol{\alpha}_{i,t}^{[r+1]}, \boldsymbol{\alpha}_{i,t+1}^{[r+1]}, \ldots, \boldsymbol{\alpha}_{i,t^*}^{[r+1]}$ together based on $\boldsymbol{\alpha}_{i,t-1}^{[r+1]}, \boldsymbol{\alpha}_{i,t^*+1}^{[r]}, \theta_i^{[r]}, \boldsymbol{\lambda}^{[r]}, \boldsymbol{X}_{i,t^*}$, and $\boldsymbol{L}_{i,t^*}$, according to Equation (7);
For $i = 1, \ldots, N$, update θ_i with a Metropolis-Hastings step. Sample $\theta_i^{[r+1]} \sim N(\theta_i^{[r]}, \sigma_\theta^*)$, accept with probability $\min\left\{1, \frac{P(\theta_i^{[r+1]} | \boldsymbol{\alpha}_i^{[r+1]}, \boldsymbol{\lambda}^{[r]})}{P(\theta_i^{[r]} | \boldsymbol{\alpha}_i^{[r]}, \boldsymbol{\lambda}^{[r]})}\right\}$, with $P(\theta_i \mid \boldsymbol{\alpha}_i, \boldsymbol{\lambda})$ in Equation (10);
For $i = 1, \ldots, N$, update $\tau_i^{[r+1]}$ based on Equations (11) given $\mathbf{a}^{[r]}, \boldsymbol{\gamma}^{[r]}, \boldsymbol{D}_i^{[r+1]}, \sigma_\tau^{2[r]}, \boldsymbol{\alpha}_i^{[r+1]}$, and $\boldsymbol{L}_i$;
Based on the conditional distribution of σ_τ^2 in Equation (12) and $\boldsymbol{\tau}^{[r+1]}$, obtain $\sigma_\tau^{2[r+1]}$;

Based on $\boldsymbol{\alpha}^{[r+1]}$, update $\boldsymbol{\pi}^{[r+1]}$ according to Equation (9);
Sample $\omega^{[r+1]}$ from the Beta distribution given in Equation (6), based on $\boldsymbol{D}^{[r+1]}$;
Update $\boldsymbol{\lambda}$ with a Metropolis-Hastings step: For $h = 0, 1, 2$, sample $\lambda_h^{[r+1]} \sim$ Uniform$(\lambda_h^{[r]}, \delta_h^*)$. Accept with probability $\frac{P(\boldsymbol{\lambda}^*|\boldsymbol{\alpha}^{[r+1]},\boldsymbol{\theta}^{[r+1]})}{P(\boldsymbol{\lambda}^{[r]}|\boldsymbol{\alpha}^{[r+1]},\boldsymbol{\theta}^{[r+1]})}$, where $\boldsymbol{\lambda}_{h\prime}^* = \boldsymbol{\lambda}^{[r+1]}$ on entries $0, \ldots, h-1$, $\boldsymbol{\lambda}_h^{h\prime} = \lambda_h^{[r+1]}$, and $\boldsymbol{\lambda}^{h\prime} = \boldsymbol{\lambda}^{[r]}$ on entries $h+1, \ldots, 3$. $P(\boldsymbol{\lambda} \mid \boldsymbol{\alpha}^{[r+1]}, \boldsymbol{\theta}^{[r+1]})$ is given in Equation (13);
For $j = 1, \ldots, J_t \times T$, sample $s_j^{[r+1]}$ from the truncated beta distribution based on Equations (14) and $\boldsymbol{\alpha}^{[r+1]}, \boldsymbol{D}^{[r+1]}, \boldsymbol{X}$, and $g_j^{[r]}$; sample $g_j^{[r+1]}$ based on $\boldsymbol{\alpha}^{[r+1]}, \boldsymbol{D}^{[r+1]}, \boldsymbol{X}$, and $s_j^{[r+1]}$;
For $j = 1, \ldots, J_t \times T$, sample $a_j^{[r+1]}$ based on Equation (16) and $\boldsymbol{L}, \boldsymbol{\tau}^{[r+1]}, \boldsymbol{\alpha}^{[r+1]}, \boldsymbol{D}^{[r+1]} \phi^{[r]}$ and $\gamma_j^{[r]}$; sample $\gamma_j^{[r+1]}$ from the normal distribution in (17), based on $\boldsymbol{L}, \boldsymbol{\tau}^{[r+1]}, \boldsymbol{\alpha}^{[r+1]}, \boldsymbol{D}^{[r+1]}, \phi^{[r]}$ and $a_j^{[r+1]}$;
Sample $\mu_1^{[r+1]}$ from the conditional distribution given in Equation (19), based on $\boldsymbol{L}, \boldsymbol{D}^{[r+1]}$, and $\sigma_1^{2[r]}$;
Sample $\sigma_1^{2[r+1]}$ based on Equation (20), given $\boldsymbol{L}, \boldsymbol{D}^{[r+1]}$ and $\mu_1^{[r+1]}$;
Sample $\phi^{[r+1]}$ from the normal distribution in Equation (18), given $\boldsymbol{D}^{[r+1]}$, $\mathbf{a}^{[r+1]}, \boldsymbol{\gamma}^{[r+1]}$, $\boldsymbol{\tau}^{[r+1]}, \boldsymbol{\alpha}^{[r+1]}$, and $\boldsymbol{L}$;

Output: Samples for each model parameter from iteration 1 to R.
